# The Potential Role of Therapeutic Drug Monitoring for Safe and Effective Anti-Infective Therapy with Manipulated Dosage Forms

**DOI:** 10.3390/pharmaceutics18020176

**Published:** 2026-01-29

**Authors:** Sara Baldelli, Fabio Borgonovo, Anastasia Foppoli, Andrea Gori, Dario Cattaneo, Matteo Cerea

**Affiliations:** 1 Pharmacology Unit, Clinical Chemistry Laboratory, ASST Spedali Civili di Brescia, 25123 Brescia, Italy; sara.baldelli@asst-spedalicivili.it; 2 Division of Infectious Diseases, Luigi Sacco Hospital, University of Milan, 20157 Milan, Italy; 3 GazzaLab, Dipartimento di Scienze Farmaceutiche, Università degli Studi di Milano, Sezione di Tecnologia e Legislazione Farmaceutiche “Maria Edvige Sangalli”, 20133 Milan, Italy; 4 ADD srl, Advanced Drug Delivery, via Paleocapa 6, 20121 Milan, Italy; 5 Department of Biomedical and Clinical Sciences, University of Milan, 20157 Milan, Italy; 6 Department of Biomedical Sciences, Humanitas University, Pieve Emanuele, 20072 Milan, Italy; 7 Clinical Analysis Laboratory, IRCCS Humanitas Research Hospital, Rozzano, 20089 Milan, Italy

**Keywords:** therapeutic drug monitoring, manipulation, anti-infectives, dysphagia, c rushing, solid dosage form, antibiotics, antiretrovirals

## Abstract

**Background:** Therapeutic drug monitoring (TDM) is essential for ensuring safe, effective, and individualized anti-infective therapy, particularly in patients with complex clinical needs. Variability in pharmacokinetics, challenges in drug administration, and high-dose regimens can compromise adherence and increase the risk of therapeutic failure or resistance. Swallowing difficulties, a common barrier to oral therapy, often necessitate alternative administration routes or customized formulations. However, interventions such as pharmaceutical compounding or manipulation of solid dosage forms may significantly alter drug bioavailability and pharmacokinetic profiles, making TDM indispensable for guiding dose adjustments and maintaining therapeutic targets. **Objectives:** This review not only emphasizes the clinical relevance of TDM but also addresses practical strategies that enable therapy when standard formulations are unsuitable or unavailable, while minimizing risks that could compromise treatment efficacy and safety. Special focus is given to anti-infective agents, such as antibiotics, antivirals, and antifungals, illustrating how TDM, combined with tailored pharmaceutical approaches, supports precision dosing and informed decision-making. **Conclusions:** Through clinical examples and pharmacokinetic considerations, we demonstrated that TDM is a cornerstone of personalized medicine, improving outcomes, and reducing adverse effects in anti-infective treatment.

## 1. Introduction

In 2014, the World Health Organization (WHO) acknowledged antibiotic resistance as the foremost threat to global health [1]. This phenomenon significantly impacts public health worldwide, with an estimated 1.91 million deaths attributable to antimicrobial resistance (AMR), which could occur globally in 2050 [2]. Although several strategies have been implemented, including the exploration of new antimicrobial agents, the use of bacteriophages or their enzymes, and the development of next-generation vaccines [3], these innovations require extensive validation before clinical use. Therefore, it is crucial to maximize current resources and minimize the misuse of antibiotics, both in healthcare facilities and within communities. In this context, the determination of drug concentrations and the application of therapeutic drug monitoring (TDM), defined as the clinical practice of measuring specific drug concentrations in biological fluids to optimize dosing, maximize efficacy, and minimize toxicity, may provide valuable support. While the adverse effects of antibiotic resistance in hospitals are well-documented (increased mortality rates, longer hospital stays, and higher healthcare costs), the clinical implications of antibiotic resistance in patients with community-acquired infections are not yet fully investigated. There is currently evidence of rising antibiotic resistance in community settings [4,5]. Since most antibiotic consumption occurs in the community, it is essential to understand the chemical and pharmacological properties of available antibiotics to implement large-scale antibiotic stewardship programs [6]

Antimicrobials are predominantly administered either intravenously (IV) or orally (PO). The IV route is primarily employed within healthcare facilities for managing acute situations that demand maximum antibiotic bioavailability in the shortest possible time. In contrast, the PO route is preferred in outpatient settings. Historically, IV therapy was considered superior; however, accumulating evidence demonstrates that appropriately selected oral regimens are non-inferior to IV therapy in many non-critical conditions [7,8].

Transitioning from IV to oral therapy can significantly enhance patient outcomes by reducing intravenous drug-related adverse effects and facilitating earlier hospital discharge. Moreover, oral therapy presents numerous advantages, including a decreased risk of cannula-related infections [9,10] and thrombophlebitis [10,11,12], as well as overall cost-effectiveness, which encompasses lower hidden costs [10,13,14]. Therefore, while both routes have their distinct applications, the emerging evidence supports the strategic use of oral antimicrobials as a viable alternative to IV therapy in appropriate clinical scenarios.

In antibiotic stewardship programs, one of the critical principles is to ensure that antibiotics are administered in a way that best suits the patient’s individual needs. This means considering factors such as the severity of the infection, the patient’s overall health, and any underlying conditions they may have. By tailoring the route of administration to the patient’s specific situation, healthcare providers can maximize the effectiveness of the treatment while minimizing potential side effects and the risk of developing antibiotic resistance. This personalized approach ensures that patients receive the most appropriate and effective care, and by better adhering to the prescription, they take the full course of the medication.

For anti-infective agents, pharmacokinetic/pharmacodynamic (PK/PD) relationships are critical for ensuring therapeutic efficacy. Beta-lactams are generally time-dependent, with efficacy best predicted by the fraction of time the drug concentration remains above the minimum inhibitory concentration (fT > MIC). Aminoglycosides and certain antivirals are concentration-dependent, where the ratio of peak concentration to MIC (C_max_/MIC) correlates with the effect. Other agents, such as fluoroquinolones and vancomycin, exhibit AUC-dependent activity, with the area under the plasma concentration–time curve relative to MIC (AUC/MIC) serving as the key predictor of efficacy. For antiviral agents, additional PK/PD indices are commonly used, including the inhibitory quotient (IQ) and protein-adjusted IC90 (PA-IC90), which help relate drug exposure to the virus susceptibility in vitro and guide optimal dosing.

When dosage forms are manipulated, drug exposure may vary, potentially affecting the ability to achieve these PK/PD targets (the manipulation of the pharmaceutical formulation may result in altered PK, thus altering the numerator and the possibility to reach or not the PK/PD targets dictated by the available literature). Recognizing the PK/PD classification of each agent helps clinicians anticipate the clinical impact of altered formulations and optimize therapy to maintain efficacy and safety.

One of the most common conditions that can significantly impact a patient’s ability to adhere to prescribed treatment is difficulty swallowing medications. Such a condition, known as dysphagia, is particularly prevalent among older adults and individuals with neurological disorders such as stroke or Parkinson’s disease. Dysphagia can also arise from structural abnormalities in the throat, such as esophageal strictures or tumors, which can obstruct the passage of tablets and capsules; psychological fears of choking causing patient throat to tense up and make swallowing difficult; or conditions that cause dry mouth. Recent meta-analysis reports oropharyngeal dysphagia prevalence at 36.5% (95% CI 29.9–43.6) in hospitals, 42.5% (95% CI 35.8–49.5) in rehabilitation centers, and 50.2% (95% CI 33.3–67.2) in nursing homes [15].

Addressing this challenge requires a personalized approach. Healthcare providers might consider alternative routes of administration, such as parenteral rectal or transdermal, or alternative medication forms, such as liquids or dissolvable tablets, to ensure that patients can take their medications safely and effectively.

**Figure 1:**
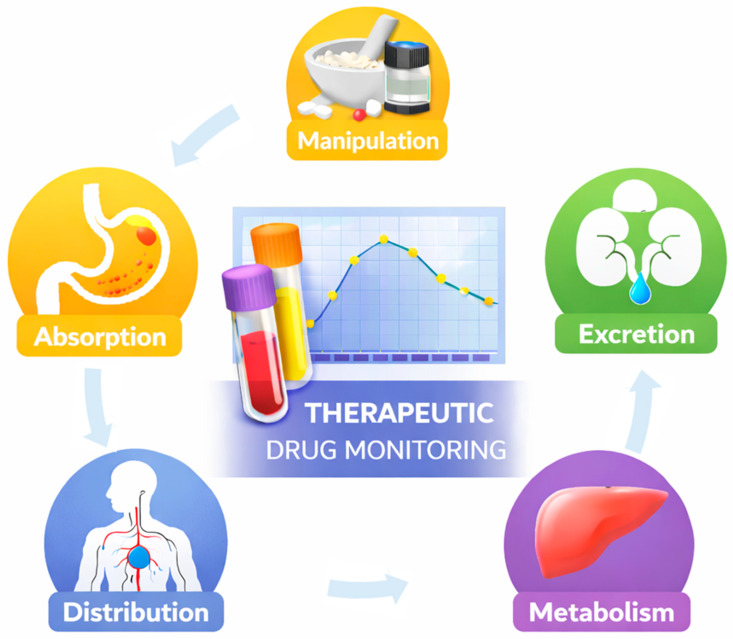
Schematic representation of the relationship between dosage form manipulation and ADME processes, showing how altered absorption can affect systemic exposure assessed through therapeutic drug monitoring (TDM).

However, there are often no alternative licensed formulations of the drug available; therefore, to make available drugs that can be administered through liquid formulations, the galenical practice of pharmacies can help to supply dosage forms in syrup, solution, or suspension of drugs made on medical prescription. However, the raw materials of the active ingredient are not always available, and a widespread practice involves crushing tablets and opening capsules, or any other solid pharmaceutical forms available on the market, for the preparation of liquid formulations. Manipulation or alteration of solid oral pharmaceutical forms can have implications on the bioavailability of the active ingredient with consequences on the efficacy and toxicity/side effects of the therapy. For example, grinding immediate-release tablets that deliver poorly soluble drugs involves an increase in the area exposed to the dissolution fluid and could potentially lead, compared to the form administered intact, to a more ready availability of the drug for absorption and therefore an increase in their bioavailability. For active ingredients with a limited therapeutic index, this could lead to risks for the patient’s health. In such scenarios, TDM can help in confirming that manipulated formulations still deliver adequate and safe drug exposure (Figure 1).

To proceed safely, a bioequivalence study between the preparation in its original form and that obtained by manipulation or reformulation would be required. Such formal bioequivalence studies are necessary for the registration of new medicinal products and would require a dedicated regulatory development program conducted by pharmaceutical companies. However, this approach is not feasible in extemporaneous or emergency clinical situations, where manipulation of the available dosage form often represents the only practical option to ensure drug administration.

Several studies have been reported in the literature in which the impact of manipulation has been rigorously investigated [16,17,18,19,20,21,22]. Some significant examples are reported relating to antibiotics, antivirals, and antifungals. Fouad and coauthors performed a Phase 1, open-label study to assess the relative bioavailability of crushed tebipenem pivoxil hydrobromide, an orally bioavailable carbapenem prodrug of the active agent tebipenem, administered through a nasogastric tube with and without enteral feeding in 12 healthy subjects [22]. Tebipenem pharmacokinetic parameters in plasma for subjects who received a crushed tablet via nasogastric tube (relative to whole tablet) and a crushed tablet with enteral feeds (relative to whole tablet) were as follows: C_max_ 11.1 ± 3.9 (12 ± 3.4) and 10.2 ± 1.9 (10 ± 4) mg/L; AUC_0–8_, 17.5 ± 3.5 (17.9 ± 2.3) and 15 ± 4.3 (13.4 ± 5.3) mg·h/L. Using the 90% CI criteria, C_max_ and AUC_0–8_ values for tebipenem were found to be bioequivalent following alternative methods of administration compared with oral dosing of the whole tablet. Since the participants were healthy, non-infected volunteers, it is not possible to draw conclusions about the potential impact on PK/PD targets.

Administration through a nasogastric tube is frequently necessary, with a solution or suspension obtainable by crushing the tablet or opening the capsule. The use of ciprofloxacin obtained from tablets for enteral feeding was studied by Druckenbrod and coauthors, who assessed in vitro the actual amount delivered through the feeding tube after suspending the drug in water [23]. Ciprofloxacin concentrations of samples delivered without and with feeding tubes were 11.77 ± 0.78 µmol/L and 12.16 ± 0.65 µmol/L, respectively. The percentage difference (−3.3%, *p* = 0.04) was considered not clinically significant. The authors concluded that administration of crushed ciprofloxacin through a small-bore feeding tube does not result in any measurable loss of drug delivered to the gastrointestinal tract.

Most antiretrovirals used for the treatment of HIV are available as oral, fixed-dose formulations (FDC) in single tablet regimens (STRs) to be taken regardless of food (with some exceptions such as rilpivirine and elvitegravir). However, there are some clinical conditions that require changes in dosage form, such as dysphagia or the need to administer the drugs through enteral feeding tubes [24]. In these cases, it is mandatory to know whether crushing or chewing the tablets can ensure an optimal systemic exposure and comparable bioavailability to marketed in-label formulations. In 2019, Roskam-Kwint et al. documented in healthy volunteers that crushing FDC tablets containing dolutegravir/abacavir/lamivudine resulted in higher dolutegravir exposure (+30%) [25]. However, the authors concluded that the FDC formulation can be crushed and administered simultaneously with enteral nutrition in patients with swallowing difficulties, as the higher dolutegravir exposure did not exceed the exposure after intake with food or in twice-daily dosing.

Brown and coauthors investigated the impact of splitting or crushing on the relative bioavailability of the darunavir/cobicistat/emtricitabine/tenofovir alafenamide STR in 30 healthy adults [26]. For the crushed versus whole tablet, the bioavailability of darunavir, cobicistat, and emtricitabine were comparable, except for a 17% decrease in emtricitabine C_max_; the relative bioavailability of tenofovir alafenamide decreased by 29% and 19% for C_max_ and AUC_last_, respectively. The authors concluded that there was no clinically relevant impact on the bioavailability of STR components when administered as a split tablet compared with a tablet swallowed whole. Administration of a crushed tablet resulted in a modest decrease in tenofovir alafenamide bioavailability that was considered not clinically relevant.

Another crossover, randomized trial in 18 healthy adults reported a significant reduction in the C_max_ and AUC of tenofovir alafenamide and emtricitabine (ranging from 9% up to 49%) but not of bictegravir after crushing the FDC [27]. The authors also showed that dissolving the tablets in water provided acceptable bioavailability of all the three drugs; therefore, this strategy may be considered when the tablet cannot be swallowed whole. Such pharmacokinetic studies carried out under standardized conditions in healthy volunteers are certainly mandatory. Equally important, however, are TDM assessments in patients with HIV in real-life settings, as described in individual case reports [28,29].

FDC-STR are available also for direct-acting antivirals used for the treatment of hepatitis C. A Phase 1 study to evaluate the effect of crushing, cutting into half, or grinding of glecaprevir/pibrentasvir tablets on systemic drug exposures in healthy subjects was carried out by Oberoi et al. [30]. Compared with the reference whole tablets, cutting into half had minimal impact on drug exposures (≤15% difference), whereas grinding or crushing the tablets resulted in lower exposures (27% to 61%) for glecaprevir and higher exposures (21% to 83%) for pibrentasvir. These results provide first guidance on appropriate administration of this STR in patients who have difficulty swallowing whole tablets. Subsequently, some case reports have been published showing the clinical effectiveness and TDM-guided optimal exposure of crushed sofosbuvir–velpatasvir treatments in patients with swallowing difficulties [31,32]. In 2020, Pijnenburg et al. investigated the influence of crushing on the pharmacokinetics of the elbasvir/grazoprevir FDC in 11 healthy adult volunteers [33]. The primary pharmacokinetic parameters AUC_0–∞_ and AUC_0–72_ of elbasvir and grazoprevir after intake of a crushed tablet were on average 12–16% higher compared with the whole tablet, but 90% CIs were all within the predefined boundaries of pharmacokinetic similarity. Crushing leads to a higher C_max_ of grazoprevir (42%); no significant difference was found between treatments regarding the C_max_ of elbasvir. Crushed and suspended administration of elbasvir/grazoprevir can be used in patients with swallowing disorders. Lastly, Huffman et al. reported the treatment of chronic hepatitis C virus infection with crushed ledipasvir/sofosbuvir administered through a percutaneous endoscopic gastrostomy tube in a patient with HIV co-infection [34]. At 12 weeks, the patient had achieved a sustained virologic response, providing clinical evidence on the usefulness of ledipasvir/sofosbuvir coformulation in crushed form. However, no pharmacokinetic data were provided. Similarly, no PK data were available on PEG-ledipasvir/sofosbuvir.

Only one study is available in the literature dealing with the comparative pharmacokinetics of voriconazole administered orally as either crushed or whole tablets in healthy volunteers [35]. In this open-label, randomized, two-way crossover comparative study, 20 subjects received voriconazole tablets either crushed or whole at the dose of 400 mg every 12 h for 1 day orally followed by 200 mg every 12 h orally for 5.5 days. The adjusted mean AUC_0–12_ for the crushed and whole tablet groups were 9793 and 11,164 ng∙h/mL, respectively (ratio, 87.72; 90% CI, 80.97, 95.04). The ratio of the maximum concentration of drug in serum for the crushed tablet versus whole tablet arms was 94.94 (90% CI, 86.51, 104.22). The only difference noted between groups was a slightly faster time to maximum concentration of drug in serum when subjects received crushed tablets, 0.5 h versus 1.5 h (90% CI, −0.75, −0.25). Based on these results, the authors concluded that administration of crushed voriconazole tablets is bioequivalent to whole-tablet administration. As the studies were conducted exclusively in healthy, non-infected volunteers, the implications of the reported PK differences for PK/PD targets cannot be inferred.

Although not directly related to the manipulation of commercially available medicinal products and despite the lack of pharmacokinetic evaluation, the work by Bass and coauthors deserves mention [36]. In their study, vancomycin for the treatment of Clostridium difficile infections was compared following administration as hard gelatin capsules versus an extemporaneously prepared oral solution obtained from powder. The study, conducted in a relatively small cohort of patients (*n* = 72), showed that efficacy, expressed as time to clinical cure, was comparable between the two formulations: there was no difference in the clinical cure at day 10 (64% solution versus 59% capsules, *p* = 0.664).

Routine integration of TDM in these contexts allows clinicians to personalize dosing, verify the success of unconventional administration methods, and prevent avoidable toxicity or treatment failure.

The aim of our review is to describe the main features that should be taken into consideration when using commercial solid drug products for the preparation of liquid compositions. The alteration of pharmaceutical forms requires adequate expertise in the formulation to understand which types of pharmaceutical form can be used, what risks are involved in the operation, and what consequences this practice could have for the patient [37,38]. In this discussion, the aspects related to the off-label use of the medicinal product will not be taken into consideration, nor the legal aspects related to the practice of handling the dosage forms, but rather the problems relating to the use, for the same route of administration, of pharmaceutical dosage forms that have been obtained by dispersion of particles in a liquid resulting from crushing an industrial product. This aspect raises a further potential issue which will only be mentioned with respect to the compatibility of the active ingredients with specific excipients. Finally, selected real-world cases illustrate how TDM-guided monitoring can support safe and effective antimicrobial therapy when dosage-form manipulation is unavoidable.

This article is conceived as a narrative review. Its purpose is not to provide a systematic appraisal of the literature, but rather to offer a qualitative synthesis of the limited, and often fragmented, evidence available on therapeutic drug monitoring (TDM) in the context of dosage-form manipulation. The scarcity of robust pharmacokinetic and clinical data, together with the heterogeneity of the scenarios in which drug manipulation occurs, makes a systematic approach neither feasible nor aligned with the objectives of this work. Instead, we aim to integrate the few published examples with relevant pharmaceutical and biopharmaceutical considerations, thereby providing a coherent framework to support clinical decision-making when manipulated medicinal products are involved.

## 2. Manipulation of Solid Oral Dosage Forms

### 2.1. Classification and Features of Dosage Forms

The preferred route for drug administration is oral, and solid oral dosage forms are the most used formulations. The advantages of this route are often precluded for patients with swallowing difficulties or unconscious patients as those in intensive care units [39,40]. For all these cases, the use of liquid dosage forms is essential, better if in solution, and even better if in viscous solution (oral gel) [41,42,43].

However, liquid pharmaceutical forms present problems of stability and, in the case of multidose products, also have issues of volume and dosage reproducibility and accuracy. A very valid alternative could be a ready-to-use powder or granules packaged in a sachet for extemporaneous preparation of suspension or solution. Technological research has also proposed oral disintegrating tablets, composite freeze-dried wafers, and films or strips, which, upon contact with salivary fluids or small volumes of fluids, generate solutions or suspensions [44,45,46,47]. These dosage forms, compared to classic solid pharmaceutical forms, offer excellent drug delivery properties, allowing the creation in situ of a swallowable solution or suspension, or the preparation of a liquid containing the drug through simple mixing with a fluid or food. However, orodispersible forms very often require an important formulation process, which presents many production and packaging problems, without neglecting the difficulty in masking the unfavorable flavor of the drug.

As a rule, the smaller the dosage form, the easier it is to swallow. In fact, tablets with a diameter < 10 mm are easier to accept, and hard capsules with a size smaller than 2 (Coni-snap^®^ format) are relatively easier to swallow. Among solid pharmaceutical forms, the oblong-shaped (biscuit-shaped) tablet is easier to swallow than a round shape of the same mass [48,49,50]. Recent studies have even suggested that the administration of minitablets with a diameter of less than 3 mm is preferred in the administration of medicines in pediatric patients compared to syrup formulations [49,50,51]. However, for a good majority of the pharmaceutical products on the market, pharmaceutical forms in solution are not available, and medicines formulated in minitablets are currently very rare.

All these problems are accentuated when patients have difficulty swallowing because they suffer from temporary and reversible dysphagia as in the case of pathologies of the oropharyngeal cavity, or permanent and irreversible as in the case of patients suffering from neurodegenerative pathologies, dementia, or muscular pathologies. In the case of uncooperative or unconscious patients or those fed through a nasogastric tube, as in the case of patients hospitalized in intensive care, the use of liquid forms of drug administration is a necessity [52,53,54]. Typically, patients in intensive care receive a large number of medications (on average, 30 different drugs during their stay), which also raises the risk of drug–drug interactions [55,56]. Furthermore, when the medicines are intended for pediatric patients under one year, by definition, liquid dosage form is essential for oral administration.

Finally, patient adherence to therapy, also referred to as compliance, should not be overlooked, particularly when oral solid dosage forms are bulky and when treatment is chronic. In such cases, poor adherence may have relevant pharmacokinetic and pharmacological consequences, as well as significant pharmacoeconomic implications associated with disease relapse [57].

Solid pharmaceutical dosage forms for oral use can be classified as conventional, also called immediate-release, in which the dissolution rate of the active ingredient depends solely on the chemical-physical characteristics of the drug, and non-conventional dosage forms, also called modified-release forms, in which the release of the active ingredient is deliberately controlled by the drug delivery system (DDS) in terms of release rate, site, or time [58,59,60]. The modified-release dosage forms are designed to obtain therapeutic objectives otherwise not achievable with conventional pharmaceutical forms. Immediate-release pharmaceutical forms can in turn be divided into coated or uncoated pharmaceutical forms. In both cases, the pharmaceutical form is not designed to control the way the active ingredient dissolves in biological fluids and is made available for absorption. The conventional solid pharmaceutical form, in contact with biological fluids, mainly aqueous, undergoes a disintegration process which releases solid drug particles for dissolution. It is not uncommon for immediate-release tablets to have incisions that allow them to be divided to adapt the dose to therapeutic needs. Tablets are often coated with soluble films that dissolve rapidly in contact with aqueous fluids. Modified-release dosage forms present a broad variety of different configurations, compositions depending on the type of release desired. Modified-release solid oral pharmaceutical forms include those having a prolonged-release, repeated-release, and gastric-resistant ones. Prolonged-release dosage forms are those designed for slowing the delivery of the drug, avoiding frequent dosing. Gastric-resistant release dosage forms, also known as delayed-release dosage forms, are prepared by applying acid-resistant coatings able to protect the coated core in the acidic gastric environment. The acid-resistant coatings consist of polymers that remain intact in the stomach but dissolve and release the ingredient active in the small intestine, where more alkaline pH is found. These formulations are used for avoiding gastric irritation (for example, NSAIDs), for protecting active ingredients that can be inactivated at gastric pH, and for delivering the active ingredient to a specific site of the gastrointestinal tract.

In this brief overview, we cannot forget the sublingual tablets, which are designed to allow a direct absorption through the oral mucosa, leading to a rapid increase in concentration of the active ingredient in the blood and thus avoiding the metabolism of first pass through the liver.

Table 1 provides a classification of the main oral pharmaceutical dosage forms. This is intended to serve as a general reference for determining cases in which manipulation should be deemed inadvisable, unless preceded by a duly conducted pharmacokinetic evaluation, in accordance with regulatory standards.

### 2.2. Practical Considerations on Crushing Oral Dosage Forms

Perhaps the most used method for crushing solid dosage forms for oral administration is performed by the patient, when chewing tablet before swallowing, often without consulting the prescribing doctor or the pharmacist. As a matter of fact, mastication allows a suspension of material in uncontrolled fragments to be generated in the buccal cavity, which is more easily swallowed.

The most traditional procedure for crushing tablets involves the use of a mortar and pestle by pharmacists, nurses, or other personnel responsible for dispensing medicines [38,39,64]. Alternatively, hammers or mallets or other hitting elements are used in combination with containers in which the solids are broken into pieces or crushed. In the mortar and pestle technique, the force applied by the operator is difficult to control, and the grinding movement is not easily standardized. Force required for breaking tablets, especially if film coated, can go up to 200 N, approximately 20 kg, with high effort for the operator. During the use of the mortar and pestle, fragments of the pharmaceutical form can involuntarily exit the container, leading to product loss and inaccurate dosing. In this respect, quantitative recovery of ground powder is essential, and frequently constitutes the main risk of inaccuracy in the dosage of the manipulated drug. The procedure also leads to the generation of volatile dusts, with potential risks for the operator in the case of particularly toxic active ingredients. The particles may cause undesired effects to the person crushing the tablets (measures must be taken to prevent skin contact and inhalation by wearing gloves and/or masks), and advice should be sought from a pharmacist. Finally, the use of the mortar and pestle technique creates loud irritating noises which often annoy the user or nearby people [64].

The use of “pill-crusher” commercially available is an option to ease the splitting and fragmentation. Different types of devices can be operated manually or electrically for crushing the tablet (pill crushers, tablet crushers, pill grinders) [65]. These devices rely on the use of blades, vibrations, pistons, screws, and combinations thereof to fragmenting the tablets down to granules or powders. Crushers can also limit the exposure of the operator to the aero-dispersed powders and the loss of fragments in the environment.

Both for the mortar and pestle and for the “pill crushers”, the fragmentation systems must be carefully cleaned and dried after each use to avoid contamination. Disposable heavy plastic bags can also be used to isolate tablets to be crushed, taking care to avoid breaking the sheet when pounding the contents, and with the attention to limit fragments or powders getting stuck in bag corners or to the plastic sheet surface due to electrostatic forces.

**Figure 2:**
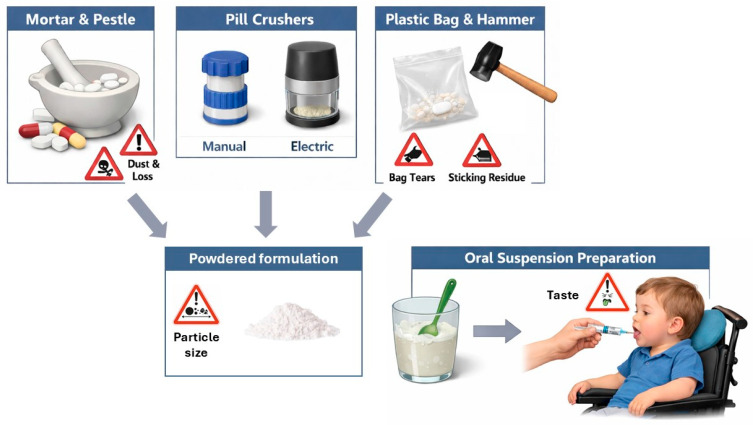
Schematic illustration of different procedures commonly employed for crushing solid dosage forms for oral administration.

Scored tablets, which present an indentation with the shape of a line or of a cross, can usually be divided into two or more pieces to allow dose adjustment. However, tablets intended for modified drug release, even if presenting scored lines, are not to be divided unless differently recommended in the product information summary reviewed by the regulatory authorities, as fragments may not deliver the same amount of drug or may alter the delivery pattern (release rate or time) achievable by the whole tablet [66]. Figure 2 depicts the different practical approaches to crushing solid dosage forms prior to their dispersion in liquids for oral administration.

### 2.3. Administration Problems

Crushed tablets are often unpalatable and may also have irritating or anesthetic effect on the oral mucosa, which can put the patient at risk [67,68,69]. Administration with liquid or rinsing the mouth with an appropriately thickened fluid after administration may help to reduce the irritation effect on the buccal cavity [70,71]. Typically, the fragmented tablets are administered in a slurry mixed with beverages or thick food to avoid separation. The texture of the suspension can ease the dosing procedure and can improve administration to patients with dysphagia, but the risk associated with a slower dissolution of the drug as compared to water should be considered. On the other hand, viscous liquids can help in masking unpleasant tastes which can enhance compliance to the therapy. Beverages or food can mitigate unpleasant taste of drug [65,71]. Specific organoleptic characteristics can be masked or reduced using different flavors often used in combination.

In pediatric patients, palatability represents a major barrier to oral drug administration, independent of swallowing difficulties or dysphagia as a medical condition. Taste aversion may significantly compromise adherence, particularly when adult-approved solid dosage forms are manipulated. However, the primary focus of the present review is on adult patients and on the pharmacokinetic and pharmacodynamic implications of dosage form manipulation. Although commercial flavoring agents and compounding vehicles are available to support pharmacists in improving palatability, a detailed discussion of pediatric formulation strategies and taste-masking approaches is beyond the scope of this work.

## 3. Case Studies

### 3.1. Isentress^®^ 400 mg Film Coated Tablets (Raltegravir)

Raltegravir is an antiviral drug used in combination with other drugs to treat adult patients infected with HIV-1. The drug is an integrase inhibitor, which is involved in HIV reproduction. When the enzyme is blocked, the virus is unable to reproduce normally, thus slowing the spread of the infection. Raltegravir may delay damage to the immune system and the onset of infections and diseases associated with AIDS [72].

Each tablet of Isentress^®^ contains 400 mg of raltegravir as potassium salt, along with typical ingredients for tableting, which include diluent, binder, surfactant, glidant, and lubricant (Table 2). The tablets also present a film prepared mainly with soluble materials, which upon contact with aqueous fluids rapidly dissolve. Therefore, the tablet was designed to release the active ingredient without controlling the release rate, and the dissolution rate would largely depend on the characteristics of the drug and not on the integrity of the pharmaceutical form.

Due to swallowing problems of elderly patients, physicians often ask if tablets could be chewed or crushed for administration. Based on these considerations, the manipulation of coated tablets would have been possible without compromising the therapeutic performance of the product. To confirm this possibility a pharmacokinetic study involving 82 patients was executed to verify the absorption of raltegravir from whole tablets against ground product [72]. The in vivo pharmacokinetics (0–4 h) of raltegravir from 69 patients receiving the drug by swallowing the whole tablet were compared to those obtained from 13 patients with HIV who chewed the tablet.

Patients taking the drug by chewing presented regular pharmacokinetic profiles (30 to 60% reduction in the coefficient of variation associated with the main pharmacokinetic parameters) and significantly higher drug absorption compared with patients taking the drug by swallowing the intact tablet (raltegravir C_max_, 5.404 ± 3.032 versus 3.128 ± 2.588 ng/mL, *p* = 0.004; AUC_0–4_, 11.634 ± 7.288 versus 7.007 ± 5.803 ng · h/mL, *p* = 0.011). In vitro analysis showed a lack of disintegration ability of raltegravir tablets and the relatively low solubility of the active ingredient in an acidic environment, which are highly affected by the gastric residence times, determining the variable and irregular pharmacokinetics of raltegravir [72]. For antiretroviral drugs, the trough concentration (C_trough_) is considered the key PK/PD index for efficacy; for raltegravir, the target is C_trough_ > 40 ng/mL. In this case, no significant differences were observed when comparing C_trough_ values between the two groups, although there was a non-significant trend toward higher concentrations in patients who took the drug by chewing.

Manipulation of Isentress^®^ tablets proved not only to enhance the compliance of the patients who might have problems with swallowing relatively large tablets, but also improved pharmacokinetics of the drug by reducing variability in raltegravir absorption (Table 3).

### 3.2. Prevymis^®^ 240 mg Film Coated Tablets (Letermovir)

Letermovir is a novel anti-cytomegalovirus (CMV) agent approved by the Food and Drug Administration for prophylaxis in adults undergoing allogeneic hematopoietic cell transplantation. Although letermovir does not yet have Food and Drug Administration approval for children, there are several reports describing its off-label use in children and adolescents undergoing hematopoietic cell transplantation [73,74,75,76,77]. The composition of the coated tablet presented typical excipients for compression, which include a diluent, binder, disintegrant, lubricant, and glidant (Table 2). The film applied is soluble, being prepared mainly with a polymer having pH-independent solubility and soluble in water, such as Hypromellose, and containing other excipients not affecting the dissolution. The dissolution rate of the drug would therefore mainly depend on the characteristics of the active ingredient and not on the integrity of the pharmaceutical form.

Even though Prevymis^®^ medicinal information leaflet clearly recommends administration of the whole tablet without dividing, crushing, or chewing, pediatricians often require changing the dosage form of letermovir for children that could not swallow relatively large tablets. It is worth noting that a liquid concentrate solution of letermovir formulated with hydroxypropyl-betacyclodextrin is also approved for the preparation of parenteral infusion [73,78].

Based on these considerations, the manipulation of Prevymis^®^ tablets could theoretically be possible. A comparison study was found in the literature where pediatric patients were treated with letermovir obtained from crushed tablets and administered via nasogastric tube in 4 of 9 patients [74]. Letermovir administration was feasible and well tolerated as CMV prophylaxis in this small cohort of pediatric patients undergoing hematopoietic stem cell transplantation. It is reasonable to assume that crushing the immediate-release tablet had only a nominal effect on the pharmacokinetic properties of letermovir, confirming the possibility of manipulation of the tablets for oral administration. In confirmation of this possibility, another clinical study on healthy male adults collected the pharmacokinetic parameters resulting from the single oral administration of 80 mg of letermovir in solution or in tablets leading to values of C_max_ 1470 (1003–1903) versus 1528 (1356–1777) ng/mL, t_max_ 1.00 (07.5–1.50) versus 1.00 (1.00–2.50) h, and AUC_0-inf_ 5367 (3667–7128) versus 5450 (4282–8908) ng · h/mL [79,80]. It should, however, be taken into account that the pharmacokinetics of letermovir could also be altered by pathologies and transplants, co-administration of drugs (e.g., cyclosporin), or severe hepatic impairment [80]. For letermovir, a classical PK/PD index has not been established, as the drug is used primarily for CMV prophylaxis rather than treatment of active infection.

### 3.3. Zyvox^®^ 600 mg Film-Coated Tablets (Linezolid)

Linezolid is a synthetic oxazolidinone antimicrobial drug effective for most of Gram-positive infections by bacteria and mycobacteria, and approved for the treatment of bacterial pneumonia, skin and skin structure infections, and vancomycin-resistant enterococcal (VRE) infections, including infections complicated by bacteremia resistant to other antibiotics. Zyvox^®^ tablet cores contain 600 mg linezolid and excipients for tableting, with a coating film prepared mainly with soluble materials (Table 2) [81]. Even if the leaflet of the drug product does not report any recommendation for the administration of the dosage form, the composition of the product suggests a conventional release of the tablet and manipulation would be possible without compromising the therapeutic efficacy of the drug. Powder for oral suspension of linezolid is also present on the market, even if not always available for use in hospital practice. According to the drug label, linezolid dosage adjustments are not needed in geriatric patients, and a conventional 600 mg twice daily dose is suggested.

However, in a pharmacokinetic study, linezolid caused trough plasma concentrations to be three times higher in elderly patients than in younger ones, exposing them to higher risks of dose-related hematological toxicity and of discontinuation of the drug [82,83]. Reduction in linezolid doses should be considered in elderly patients at low risk of treatment failure, ideally guided by TDM. To verify the impact of splitting Zyvox^®^ tablets, a pharmacokinetic study in 33 tuberculosis patients was performed: 13 patients were given linezolid at the standard dose (600 mg dose), while 20 patients were given half tablet (300 mg dose). The pharmacokinetic profiles were comparable, and the main PK parameters normalized with the dose were not statistically different (C_max_ 0.019 ± 0.006 versus 0.022 ± 0.01 mg/mL/mg, *p* = 0.272; AUC_0–5_ 0.0744 ± 0.036 versus 0.0739 ± 0.029 mg h/mL·mg, *p* = 0.962). This confirmed that drug release was not altered by splitting of the tablet, and, therefore, splitting can be used to provide dose flexibility and cost savings [84].

From a PK/PD point of view, linezolid can be considered both a fT > MIC- and AUC/MIC-dependent antibiotic. In the previously reported studies [83,84,85], all patients requiring linezolid dose-reduction reached and maintained the PK/PD target of C_trough_ > 2 mg/L or >1 mg/L set, respectively, as the MIC breakpoint for bacterial and mycobacterial infections.

**Table 2:** Excipient compositions of Isentress^®^ 400 mg, Prevymis^®^ 240 mg, and Zyvox^®^ 600 mg as reported in summary of product characteristics [85].

Isentress^®^ 400 mg Film Coated Tablets	Prevymis^®^ 240 mg Film Coated Tablets	Zyvox^®^ 600 mg Film-Coated Tablets
*Tablet core* Microcrystalline cellulose (E460) Lactose monohydrate Calcium phosphate dibasic anhydrous Hypromellose 2208 Poloxamer 407 Sodium stearyl fumarate Magnesium stearate	*Tablet core* Microcrystalline cellulose (E460) Croscarmellose sodium (E468) Povidone (E1201) Colloidal anhydrous silica (E551) Magnesium stearate (E470b)	*Tablet core* Maize starch (corn derived) Microcrystalline cellulose (E460) Hydroxypropylcellulose (E463) Sodium starch glycollate type A Magnesium stearate (E572)
*Film coat* Polyvinyl alcohol Titanium dioxide Polyethylene glycol 3350 Talc Red iron oxide Black iron oxide	*Film-coat* Lactose monohydrate Hypromellose (E464) Titanium dioxide (E171) Triacetin (E1518) Iron oxide yellow (E172) Carnauba wax (E903)	*Film coat* Opadry, white, YS-1-18202-A(e) comprising: Hypromellose (E464) Titanium dioxide (E171) Macrogol 400 Carnauba wax (E903)

**Table 3:** Summary of main results of clinical case studies presented on manipulation of oral solid dosage forms and pharmacokinetic implications.

Drug (Product)	Dosage Form and Release Characteristics	Type of Manipulation	Key Pharmacokinetic Findings	Clinical Implications	Role of TDM
Raltegravir (Isentress^®^ 400 mg) [72]	Film-coated tablet; immediate-release; soluble film, low solubility in acidic environment	Chewing/ grinding	Chewed tablets compared to intact tablets: C_max_ (5404 ± 3032 vs. 3128 ± 2588 ng/mL, *p* = 0.004) and AUC_0–4_ (11.634 ± 7.288 versus 7.007 ± 5.803 ng h/mL, *p* = 0.011) and reduced PK variability	Improved patient compliance and more consistent absorption, despite lack of disintegration of intact tablets	Manipulation did not compromise target attainment and reduced variability
Letermovir (Prevymis^®^ 240 mg) [79]	Film-coated tablet; immediate-release; pH-independent soluble film	Crushing and administration via nasogastric tube (pediatrics)	Crushed tablets compared to intact tablets: C_max_ 1470 (1003–1903) vs. 1528 (1356–1777) ng/mL, T_max_ 1.00 (0.75–1.50) vs. 1.00 (1.00–2.50) h and AUC 5367 (3667–7128) versus 5450 (4282–8908) ng · h/mL	Manipulation feasible for patients unable to swallow tablets; useful in pediatric and transplant settings	TDM potentially useful in complex clinical settings (e.g., drug interactions, hepatic impairment)
Linezolid (Zyvox^®^ 600 mg) [84]	Film-coated tablet; conventional release; soluble coating	Tablet splitting (300 mg vs. 600 mg)	Whole tablets compared to split tablets: C_max_ 0.019 ± 0.006 vs. 0.022 ± 0.01 mg/mL/mg, *p* = 0.272; AUC_0–5_ 0.0744 ± 0.036 vs. 0.0739 ± 0.029 mg h/mL·mg, *p* = 0.962)	Enables dose reduction, flexibility and cost savings, particularly in elderly patients	TDM-guided dose reduction ensured maintenance of C_trough_ targets and reduced toxicity risk

## 4. Limitations

Here, we aimed to describe pharmacokinetic evaluations performed in real-life clinical practice through the application of TDM, which were used to assess whether manipulations of pharmaceutical formulations, implemented to address specific clinical needs, resulted in clinically relevant changes in systemic drug exposure.

This narrative review is subject to certain important limitations, including the scarcity of robust pharmacokinetic data on manipulated dosage forms, which are derived almost exclusively from studies involving healthy volunteers, and the lack of information on formulation stability. For these reasons, the recommendations provided in this review should be regarded as an expert opinion, primarily reflecting a shared consensus among specialists in chemistry, pharmaceutics, and pharmacology, rather than the result of a systematic evaluation of the literature.

Furthermore, the reported cases are heterogeneous. Nevertheless, they originate from real-life clinical practice and from our experience accumulated over the years. Each clinical case arose from a specific, well-defined clinical need, which we addressed by tackling chemical, pharmaceutical, and pharmacological challenges, with the aim of supporting clinicians in the management of these complex scenarios.

While TDM is widely applied in clinical practice and supported by the substantial literature, this review does not provide direct outcome data for every drug or clinical scenario discussed. Therefore, recommendations regarding TDM should be interpreted as informed expert guidance rather than evidence derived from controlled clinical trials.

## 5. Conclusions

The manipulation of solid oral dosage forms of anti-infective agents can be effectively integrated into a comprehensive outpatient antimicrobial stewardship program. This approach facilitates the management of community-acquired infections in patients with dysphagia, reduces hospital admissions, and supports an appropriate transition from intravenous to oral therapy, thereby limiting intravenous-related adverse events, shortening hospital stays, and reducing healthcare costs, particularly in vulnerable patient populations.

Before proceeding with dosage form manipulation, consultation with key stakeholders (pharmacists, pharmacologists, chemists) is essential to evaluate formulation characteristics and ensure proper functionality, minimizing therapeutic failure or toxicological risk. When supportive literature data are lacking, TDM may represent a valuable tool to guide dose adjustment. From an ethical perspective, although the risks associated with drug manipulation cannot be completely excluded, practices such as crushing, splitting, chewing, or opening dosage forms may constitute a reasonable and sometimes lifesaving option when no alternative formulations or treatments are available.

**Figure 3:**
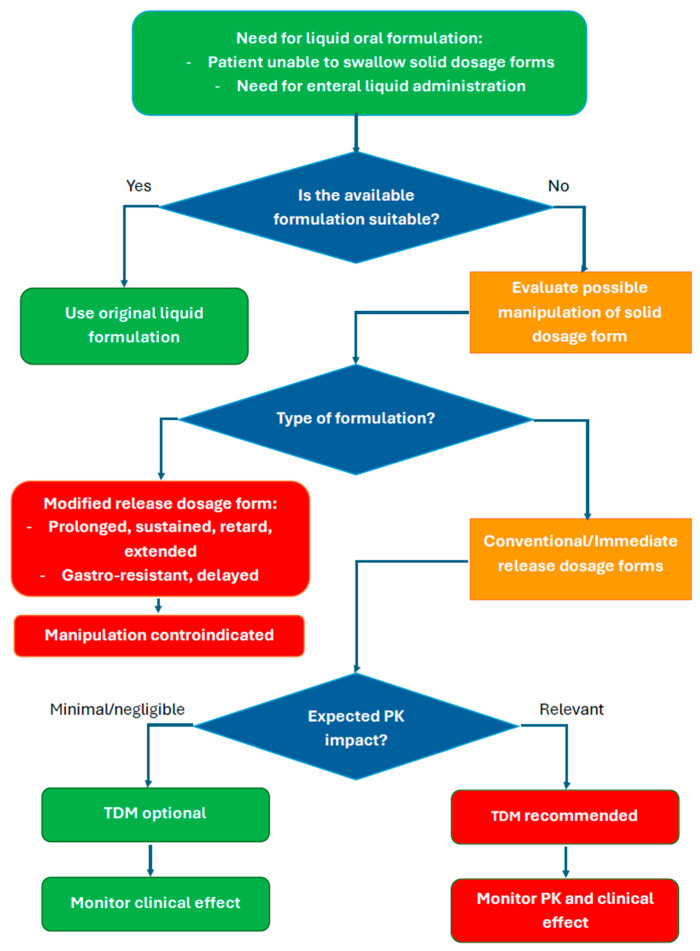
Decision-making flowchart for evaluating the need for TDM following oral dosage form manipulation.

A schematic decision flowchart illustrating when dosage form manipulation may be acceptable, contraindicated, or when TDM should be considered is given in Figure 3. Manipulation of dosage forms, especially immediate- versus modified-release formulations, may affect pharmacokinetic and pharmacodynamic parameters such as C_max_, T_max_, and the achievement of PK/PD targets, potentially influencing efficacy and safety. In this context, TDM can help mitigate uncertainty related to altered drug exposure.

However, important knowledge gaps remain. Available pharmacokinetic data are scarce, largely derived from studies in healthy volunteers, and information on formulation stability and real-world PK/PD target attainment after manipulation is limited. Future research should therefore focus on bioequivalence studies following dosage form manipulation and on real-world investigations guided by TDM to better define efficacy, safety, and clinical outcomes in routine practice.

## Figures and Tables

**Table 1:** Classification of the main oral dosage forms based on their suitability for manipulation [61,62,63,64]. The last column of the table is intended as an expert opinion, primarily reflecting a shared consensus among experts in the fields of chemistry, pharmaceutics, and pharmacology.

Dosage Form	Drug Release Mode	Details		Examples of Typical Excipients Used	Manipulation (Crushing/ Chewing)
**Uncoated tablet**	Immediate-release	Uncoated powder/granules	*Tablet*	Diluent (cellulose microcrystalline, mannitol, lactose, starch), disintegrant (cross-caramellose, cross-povidone), lubricant (magnesium stearate), anti-sticking (talc), glidant (colloidal silica), surfactant (sodium lauryl sulphate)	Ok
	Modified-release: prolonged (sustained, retard, extended, pulsatile)	Uncoated powder/uncoated granules/coated or uncoated tablets	*Tablet*	Hydrophilic polymer (hypromellose, hydroxypropyl cellulose), hydrophobic materials (ethylcellulose, stearic acid, lipids)	No
**Coated tablet**	Immediate-release	Soluble coating	*Coating*	Hypromellose (HPMC), PVA, PVP, PVP-PVA…	Ok
	Modified-release: gastro-resistance (delayed, enteric)	Gastric-resistant coating	*Coating*	pH-dependent solubility materials (methacrylate or methacrylic acid polymers, Eudragit L, HPMC-AS)	No
	Modified-release: prolonged (sustained, retard, extended)	Osmotic pump	*Coating*	Cellulose acetate	No
	Modified-release: prolonged (sustained, retard, extended)	Coated tablet	*Coating*	Ethylcellulose, Eudragit RL or RS	No
**Capsule**	Immediate-release	Powder		Diluent (cellulose microcrystalline, mannitol, lactose, starch), disintegrant (cross-caramellose, cross-povidone), lubricant (magnesium stearate), anti-sticking (talc), glidant (colloidal silica), surfactant (sodium lauryl sulphate)	Ok
		Uncoated granules		Excipients for powder formulation and binders (PVP, hypromellose)	Ok
		Coated granules	*Coating*	Soluble materials (hypromellose, HPMC, PVA, PVP, PVP-PVA)	Ok
	Modified-release	Coated granules	*Coating*	Methacrylate or methacrylic acid polymers (e.g., HPMC-AS)	No
		Pellets (uncoated/coated)	*Core*	Excipients for pelletisation and binders (MCC, PVP, hypromellose)	No
			*Coating.*	Methacrylate or methacrylic acid polymers (e.g., HPMC-AS) Hydrophilic polymer (hypromellose, hydroxypropyl cellulose), hydrophobic materials (ethylcellulose, stearic acid, lipids)	

## Data Availability

The data that support the findings of this study are available on request from the corresponding author.

## Abbreviations

The following abbreviations are used in this manuscript:

AUC	Area under the curve
CI	Confidence Interval
C_max_	Peak plasma concentration
CMV	Cytomegalovirus
FDC	Fixed-dose combination
HIV	Human Immunodeficiency Virus
HPMC	Hydroxypropyl methyl cellulose
HPMC-AS	Hydroxypropyl methyl cellulose Acetate Succinate
IV	Intravenous
MCC	Microcrystalline cellulose
PO	Oral
PVA	Polyvinyl alcohol
PVP	Polyvinyl pyrrolidone
STR	Single tablet regimen
TDM	Therapeutic Drug Monitoring
T_max_	Time to reach C_max_
WHO	World Health Organization
